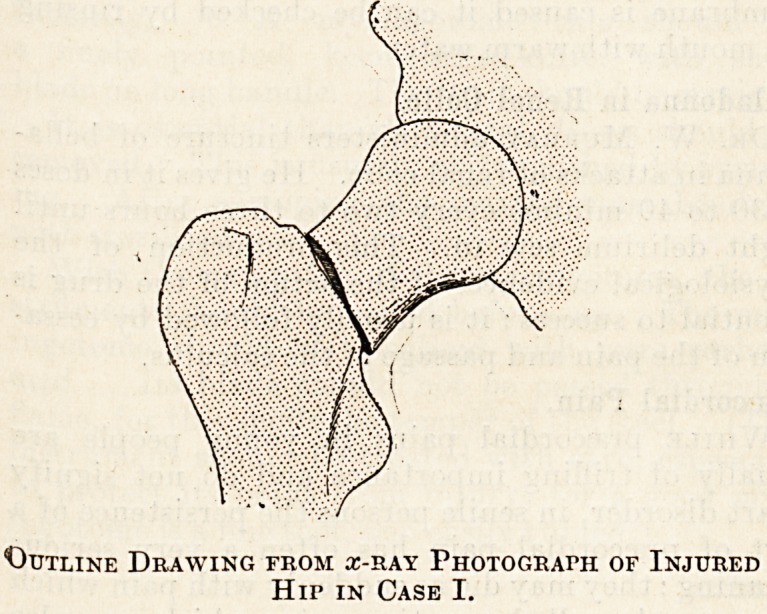# Sprains: Their Examination and Treatment

**Published:** 1907-05-11

**Authors:** 


### Points in Surgery.

### SPRAINS: THEIR EXAMINATION AND TREATMENT.

By the term "sprain" is commonly meant an injury to a joint, unaccompanied by fracture or dislocation of the bones entering into the formation of the joint. It is essential to bear in mind this negative purport of the word, and to use the term with great discretion. For if there is one mistake which the layman cannot forget or forgive, it is the overlooking by his medical attendant of a fracture or a dislocation. This is the first point. Accordingly, an injury must not be diagnosed as a sprain until every available method of examination has been applied.

Apart from medico-legal considerations, it is for therapeutic purposes highly important to discover exactly the lesion which has been produced by trauma to a joint. For if there be a fracture of bone communicating with the injured joint, too early and too vigorous movements may lead to destructive arthritis and may produce permanent crippling or deformity.

Outline Drawing from $x$-ray Photograph of Injured Hip in Case I.

Yet, in spite of these two great reasons for discretion, and with the bitter memories which must lurk somewhere in the consciousness of nearly every practitioner, mistakes are still made, and cases are repeatedly diagnosed as sprains where fractures could have been shown by the $x$-rays. The following two cases exemplify this theme:—

Case I.—The patient, aged 19 years, fell from a height of 20 ft. and hurt his right hip. On examination there was neither bruising nor deformity to be discovered in the region of the injured part. No crepitus could be elicited, and although movements of the right hip joint caused pain and were limited in extent, yet in carrying them out one did not get the impression than there was any fracture. On measuring the two lower limbs there was found to be one quarter of an inch shortening on the injured side. In the absence, however, of other definite signs these measurements were not regarded as proving that the bone was broken. But, as a precaution, an $x$-ray photograph was taken, and in this way a fracture through the neck of the femur was detected (see illustration). Unfortunately, we do not know the ultimate result of treatment in this case, although it was probably satisfactory. In any event the patient would have been unable to attribute any evil after-effects to medical negligence, as he might have done if an $x$-ray photograph had not been taken. For without the photograph the case almost certainly would not have been regarded or treated as one of fracture.

Case II.—The following case, as regards the nature of the injury, is in some respects like the foregoing. The patient, a man of about 30 years, endeavoured to get on to a vehicle in motion, but slipped and fell in the road, striking his left hip. On examination neither bruising nor deformity could be detected about the injured hip; there was no shortening of the limb, and although movements caused pain, yet within limits he permitted the thigh to be moved and could himself raise the knee from the bed. No crepitus was detected. The fact that he was very neurotic and yet, in spite of his nervous excitability, would allow movements at the affected joint, dissipated the last suspicion that a fracture might be present. As not infrequently happens, it was almost, if not quite, impossible to obtain an $x$-ray photograph in this case. The patient could not be moved to an $x$-ray apparatus, nor were there the means of bringing such an apparatus to the patient. Taking into consideration the absence of signs of fracture, the neurotic character of the patient, and the exaggeration of symptoms that so often precedes an action for compensation, this patient, after a few days' rest, was advised to start movements of the injured limb, and at the end of 11 days, in spite of his protestations against the severity of the treatment, was exhorted to get about on crutches. The patient, being now at liberty, immediately had a skiagram taken, which showed a fracture almost identical in appearance with that shown in the illustration of the preceding case. Two or three months later the patient, who in the meantime had changed his medical adviser, was again seen. He now had his left hip ankylosed in a position of marked flexion. There was considerable wasting of the thigh muscles, and an $x$-ray photograph showed the hip joint practically destroyed by osteoarthritis. Whether by the prolonged fixation and rest, which would have followed a correct diagnosis, this lamentable result might have been avoided, may be left to speculation. The point is that the failure of treatment followed a mistaken diagnosis, a combination which is apt to be peculiarly inconvenient for the medical practitioner.

It will be observed that although the present article is entitled "Sprains," yet the two illustrative cases are both instances of fracture. This apparent discrepancy was purposely designed in order to lend more forceful demonstration to the moral, that severe "sprains" are often, perhaps usually, instances of fracture, and should be treated as such unless use has been made of every method of inquiry, including the *x*-rays, by which the presence of a broken bone can be excluded. If it is impossible to make use of the *x*-rays, the administration of an anæsthetic may facilitate the diagnosis. In all cases of doubt it will be wise to treat the injured joint gently, as though there were a fracture in its vicinity, and to refrain from telling the patient that he has only got a bad sprain.

## Figures and Tables

**Figure f1:**